# Menstrual Cycle Phase, Hormonal Contraception, and Alcohol Consumption in Premenopausal Females: A Systematic Review

**DOI:** 10.3389/fgwh.2021.745263

**Published:** 2021-10-12

**Authors:** Jasmine G. Warren, Victoria M. Fallon, Laura Goodwin, Suzanne H. Gage, Abigail K. Rose

**Affiliations:** ^1^Department of Psychology, Institute of Population Health, University of Liverpool, Liverpool, United Kingdom; ^2^Liverpool Centre for Alcohol Research, University of Liverpool, Liverpool, United Kingdom

**Keywords:** menstrual cycle, hormonal contraception, alcohol, alcohol consumption, PMS

## Abstract

Women may be particularly vulnerable to alcohol harm, but many current theories fail to acknowledge the unique factors that influence female alcohol use. The biological mechanisms underlying female alcohol consumption have largely been unexplored, although recently the menstrual cycle has been highlighted as a potentially important factor. This systematic review, using a narrative synthesis, examined the association between the menstrual cycle phases on alcohol consumption and aimed to determine whether hormonal contraception influences this association. The review follows PRISMA and SWiM guidelines, registration number: CRD42018112744. Electronic searches were conducted in the relevant databases with keyword (e.g., “menstrua^*^”; “alcohol”). Thousand six hundred and sixty-two titles were identified, 16 of which were included in the review. Results were inconsistent regarding whether an association between menstrual cycle phase and alcohol consumption was found. Furthermore, there was inconsistency regarding which phase was associated with higher consumption, and different factors were reported to have moderated the direction, e.g., family history of alcohol use disorder (AUD), premenstrual syndrome (PMS). These conflicting results may be partly explained by variability in both study quality and design, and differences in measurement of cycle phase and alcohol consumption. More robust research is needed before conclusions can be drawn with regard to the role of the menstrual cycle and hormonal contraception on female drinking behavior. This review provides recommendations to strengthen research in this area.

## Introduction

Alcohol is the most used recreational substance within the UK (1). Previous research has highlighted biological sex differences in patterns of alcohol use. For example, females are more likely to drink to cope with stressors relative to their male counterparts, whereas males are more likely to drink to enhance social interactions (2). Females also progress to dependence more rapidly than males (3, 4). The literature exploring why these sex differences may exist is scarce, however, reviews have indicated a potential role of gonadal hormones and in particular the menstrual cycle (4, 5).

**Figure 1:**
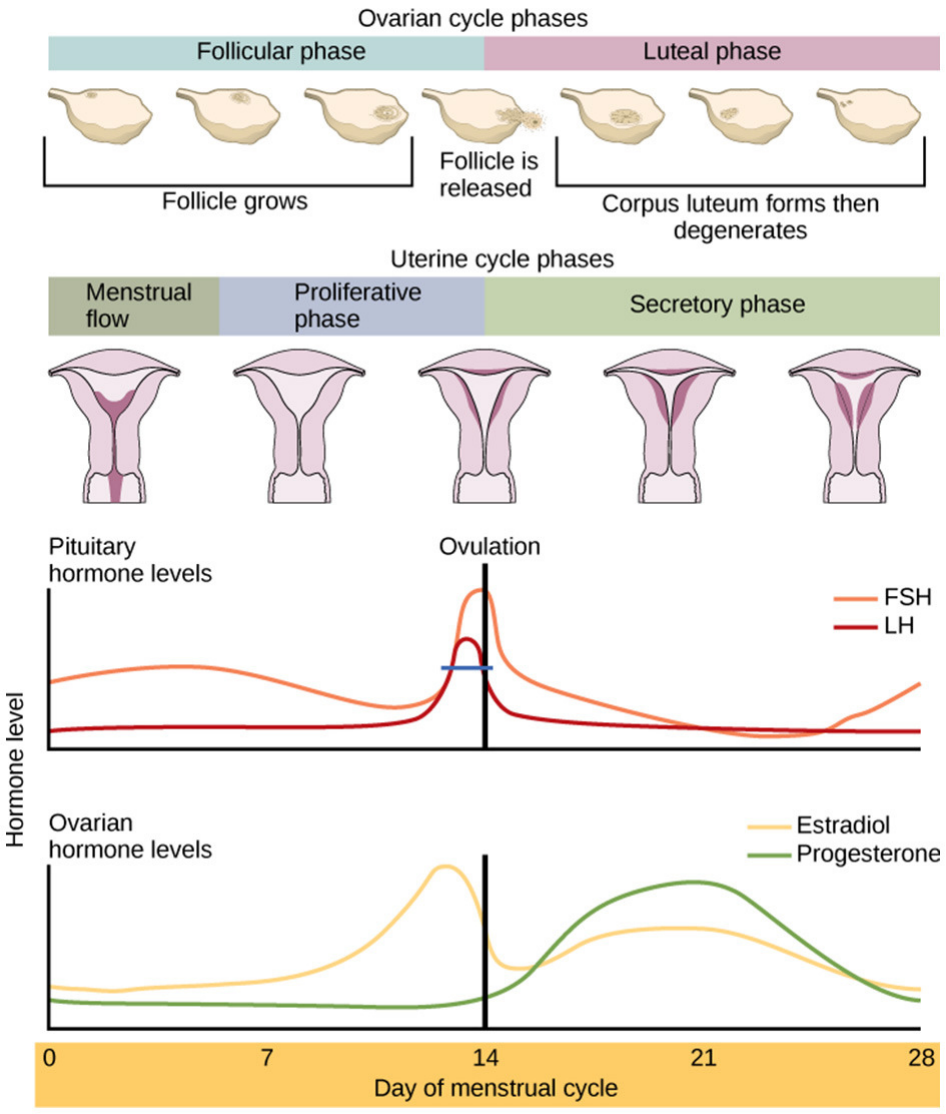
The hormonal composition of the menstrual cycle and its phases. This includes, the egg cycle, LH, FSH, Oestrogen (Estradiol [E2]), Progesterone and the lining of the uterus (7).

A typical menstrual cycle for healthy, reproductive females has two predominant phases: the Follicular Phase (FP; first day of menses to ovulation) which is, on average, 1 to 2 weeks in length. The FP encompases the sub-phases: menses, post-menstrual and ovulation. The second predominant phase is the Luteal Phase (LP; post-ovulation to the day before the next menses) which is, on average, 2 weeks in length, and encompases the premenstrual period. Although typical, there can be considerable variation in the phase lengths between healthy women and across any individual woman's cycle. Each phase is associated with specific levels and ratios of estrogens (Estrone; Estradiol; and Estriol) and progesterone. During the FP (which is usually between 1 and 2 weeks long), estrogens rise and peak just before ovulation, and while progesterone also increases and falls during this time it remains low compared to the levels of estrogens. During the LP (~2 weeks long), the levels of estrogens start to decrease whilst progesterone rises and peaks half way through the LP. estrogens also rise and fall during the LP, but levels are low relative to progesterone (6) (see Figure 1). Due to the phase specific hormonal activity and that phase length can fluctuate, research assessing the relationship between menstrual hormones levels and behavior should include biological assessments.

There is evidence that estrogens and progesterone can influence alcohol intake (4, 8). Animal studies demonstrate that higher levels of estrogens can increase neural excitability and synaptic transmission, and the metabolism rate of ethanol (9, 10). These underlying biological mechanisms may help explain why human females find alcohol more rewarding during the FP relative to the LP when levels of estrogens are highest (3, 9). Higher levels of estrogens can also increase the risk of relapse to drinking during recovery (11, 12). An additional point of consideration is hormonal contraception. The range of hormonal contraceptives fall under two general types: combined (contains both estrogens and progesterone) and progesterone-only (13). The administration of progesterone during the FP can inhibit the positive subjective ratings of substances (14), indicating that alcohol use may differ between women taking hormonal contraception and those naturally cycling.

In the field of health, approximately 500 menstrual-cycle related articles are published each year (15), however only a limited number investigate whether the menstrual cycle influences alcohol use. A recent systematic review explored the role of gonadal hormones on alcohol behavior in both humans and animals (16). The review yielded 50 articles (including animal studies and clinical samples) and, in-line with the existing literature, found that increased levels of estrogens was positively associated with alcohol consumption in animal and clinical female samples, however the role of progesterone remained unclear (16). The review considered gonadal hormones (e.g., testosterone, progesterone, estrogens) exclusively, without considering how these hormones map onto the menstrual cycle. Yet they did report that there was preliminary evidence that levels of estrogens may affect alcohol use, and a need to investigate the role of the menstrual cycle in drinking behavior.

Another meta-analysis investigated the role of the human menstrual cycle in health-related risks, including alcohol and tobacco use (17). No variability was found across the cycle with regard to alcohol and tobacco use. However, this may have been due to heterogeneity in how results were reported in the included studies, making meta-analysis inappropriate. Additionally, the only drinking-related search term used to identify papers was “alcohol,” which may have resulted in missing some of the relevant literature.

This is the first systematic review which aims to determine the association of menstrual cycle phase with alcohol use in exclusively human, non-clinical samples. It considers research with individuals who are naturally-cycling (NC) and those who have also included a comparison group of hormonal-contraceptive (HC) users, distinguishing between these categories. Through narrative synthesis this review will consider the heterogeneity in methods used and as such an additional aim is to provide methodological guidance for standardizing future research in this field.

## Methods

This review was preregistered with PROSPERO on the 23^rd^ October 2018 (https://www.crd.york.ac.uk/prospero/display_record.php?ID=CRD42018112744). Due to the studies showing high heterogeneity in their methodological approaches, we employed a narrative synthesis methodology (18). The study characteristics including the sample, design, and measures, were assessed. Studies were described according to Synthesis without meta-analysis (SWiM) guidelines (19).

### Eligibility Criteria

Peer reviewed articles and reviews were included if there was an English version of the article. Diary and experimental studies were both included if they had a human sample, with participants aged 18–40 years, this was to ensure the sample had started menstruating and before they were menopausal. Studies with pregnant samples, or menstrual irregularities/abnormalities were excluded as these affect the menstrual cycle. Additionally, those with alcohol use disorders (AUDs) were excluded as they may be less likely to have cycle variation. See Table 1 for criteria.

**Table 1:** Screening criteria.

**Inclusion criteria**	
Publication language	• English/English translation
Journal type	• Peer reviewed articles/Reviews
Types of studies	• Diary • Experimental
Participants	• Human • Aged 18–40 • Premenopausal
Independent variable	• Menstrual cycle phase • Contraception use
Outcome variable/measures	• Alcohol use: quantity/frequency of consumption
**Exclusion criteria**	
	• Current/history of alcohol or drug misuse
	• Diagnosis of PMDD

### Literature Search Strategy

Following scoping searches (see Appendix), two sets of search terms were used to identify articles published in English: menstrual cycle (Menstrua^*^, Menses, Menorrhea, Menarche) and alcohol (alcohol, ethanol, drink^*^) with Boolean operators “and” between the two sets and “or” between terms in the same set e.g., (Menstrual^*^ OR Menses) AND (alcohol OR drink^*^). The top three databases (those which yielded the highest number of search results) using the search term were: MEDLINE, Science Citation Index, and Academic Search Complete. The search was then limited to academic journals, reports, dissertations/theses, conference materials, electronic resources, and reviews. After excluding irrelevant articles by type (i.e., breast neoplasms, menopause, pregnancy, men) and removing duplicates the search yielded 1,662 results. Reviews were included until full text screening to check reference lists for any additional relevant articles. All reference lists of the included articles were also searched for relevant titles. The initial search was conducted during January 2019. JGW conducted the search again using the same term and databases in August 2021 to ensure any articles published since January 2019 were identified and screened.

### Article Selection

Two authors (JGW and VMF) conducted all stages of the three-stage screening process. First, titles were assessed to remove those unsuitable or those without an English translation. Abstracts were screened against the inclusion and exclusion criteria. Finally, the full texts were screened for eligibility. See Table 1 for eligibility criteria.

**Figure 2:**
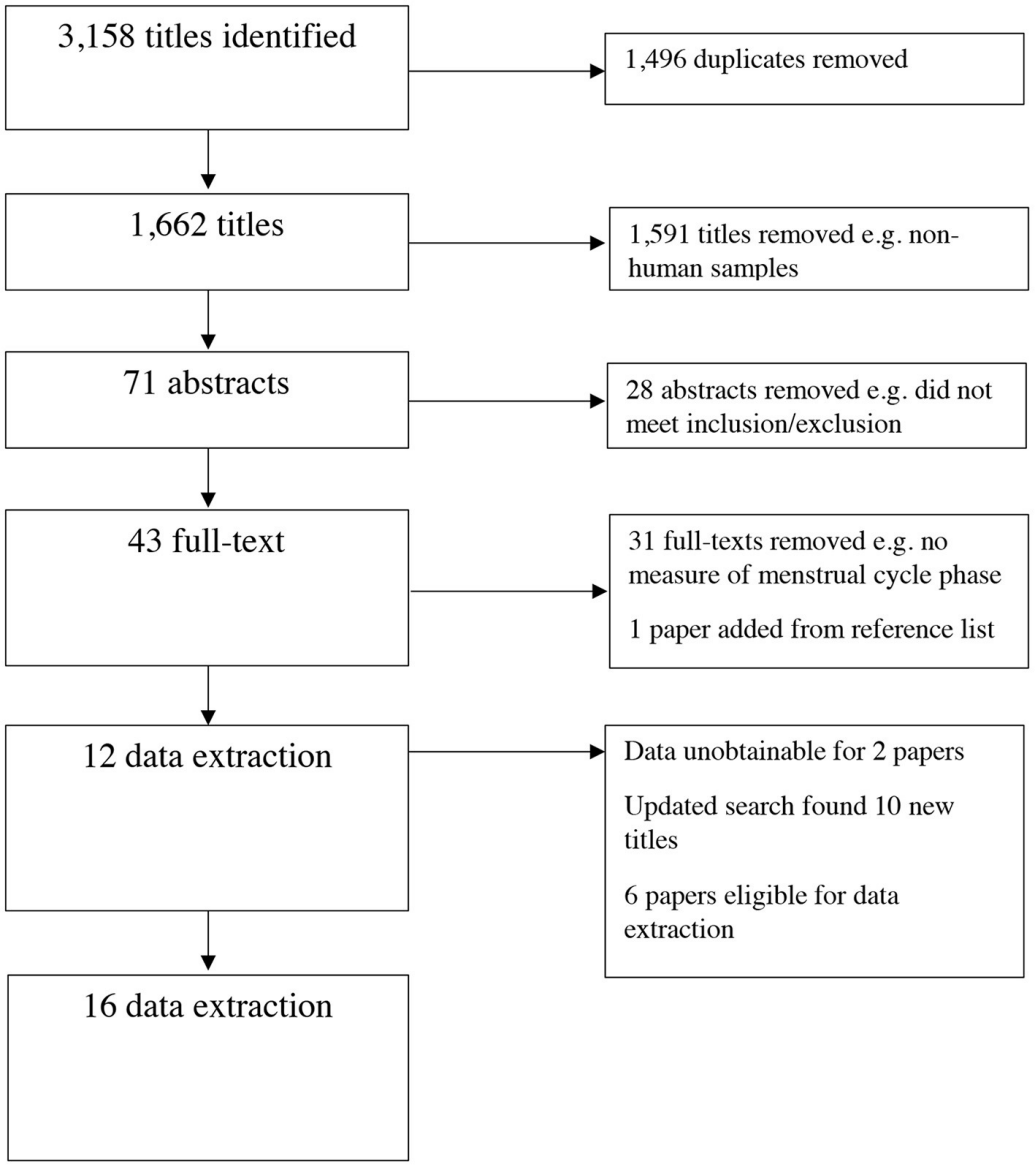
PRISMA flow chart.

Guidelines of the Preferred Reporting Items for Systematic Reviews and Meta-analyses [PRISMA; (20)] were followed and two researchers (JGW and VMF) independently screened the results. JGW screened all titles and VMF screened approximately 10%, resulting in kappa = 0.96 with consensus reached for seven titles for which the researchers were not initially in agreement. JGW screened all abstracts and VMF screened approximately 40%, resulting in kappa = 0.78 with consensus reached for the remaining four abstracts. JGW screened all full texts and VMF screened approximately 20%, resulting in kappa = 1. Full text reference sections were assessed for additional relevant articles and the authors of the included papers were contacted with a request for any additional unpublished data. Out of 12 papers, three authors were not contactable (retired/deceased). Five of the remaining nine authors responded and reported no unpublished/additional research had been conducted. Two out of 12 studies did not report the results of the association between the menstrual cycle and alcohol use, so the authors were contacted for additional information. One responded and stated the data was no longer accessible. As such, these were both removed. This resulted in 10 articles for data extraction. Six additional papers were identified for full-text screening in January 2021, all were eligible for data extraction. See Figure 2 for PRISMA flow-chart.

### Data Extraction

Two review authors (JGW and VMF) extracted the all data independently with author one screening all and author two screening 50%, yielding a kappa value of 0.86 with a consensus reached for the remainder through discussion. The information collected included study design, participant information (sample size and characteristics), methods for menstrual cycle phase determination, outcome measures, and the results. The Newcastle-Ottawa Quality Assessment Scale, which has been validated for use in women's health research (21), was used to extract data from the included papers and assess the risk of bias. Review author one extracted data from all the papers, and review author two from 50%, yielding a kappa value of 0.70. After discussion the authors reached agreement with a kappa value of 0.90. This tool has established both inter-rater reliability and content validity in previous reviews of women's health studies (21) and the Cochrane Collaboration recommends this quality assessment tool for systematic reviews of observational studies (22). As the studies varied in their: overall design, measurements of menstrual cycle phase, outcome alcohol measures and samples, a narrative synthesis was deemed the most suitable method of combining study results. The ROBIS tool was used to assess the risk of bias in the systematic review (23). As the methods and measures of the studies varied greatly, the most appropriate method of synthesis was by research design (24). This is due to heterogeneity in the overall methodological designs, with all studies falling under one of three types: lab-based, diary-based, and mixed-methods.

## Results

See Table 2 for study characteristics and findings in brief. The present results are reported according to the methods used to investigate the relationship between the menstrual cycle and alcohol. Out of the studies included in the review, only two out of the 16 reported effect sizes. JGW contacted all authors requesting effect sizes or the data. The authors of five studies were previously unobtainable and four studies did not respond. Out of the remaining five, all responded reporting they no longer had access to the data.

**Table 2:** Study characteristics.

**Reference**	**Design (duration)**	**Sample**				**Cycle phase determination**	**Alcohol use measure**	**Results**	**Additional findings**
		**Country**	N^*^ (M age)	**Characteristics**	**HC group**				
Brown et al. (25)	Diary (NR)	Hawaii	89 (21.6)	Healthy, 45 heterosexual; 21 gay; 33 sexually abstinent	–	Countback method; Ovulation test	Number of alcoholic drinks (0–5+)	No main effect of phase on consumption	Heterosexuals increased consumption from FP to LP, gay individuals decreased consumption from FP to LP
Caan et al. (26)	Diary (1 month)	USA	204 (NR)	Healthy, 76 mod-severe PMS; 26 mild PMS; 102 control	–	Diary of symptoms	24-h dietary recall (three during each phase)	More alcohol consumed for PMS group in FP compared to LP, less consumed in FP compared to LP for non-PMS	PMS group consumed more alcohol overall compared to non-PMS
Carroll (27)	Mixed (1 month)	USA	33 (23)	Healthy	–	Ovulation test	Time Line Follow Back	No main effect of phase on consumption	Moderate influence of phase on impulsivity (sensation seeking only)
Charette et al. (28)	Diary (9 week)	Canada	80 (21)	Healthy 30 high-risk of AUD; 52 low-risk of AUD	–	Diary of symptoms	Ounces (diary: number and type of alcoholic drinks)	No main effect of phase on consumption	
Christensen et al. (29)	Diary (2–3 month)	Australia	43 (33.6)	Healthy, 13 high-PMS; 17 low-PMS; 13 control	–	Diary of symptoms	Diary (average number of drinks per phase)	PMS groups had higher consumption in the LP compared to FP, no effect for the control group	No interaction with sexual activity or physical activity
Dumas et al. (30)	Diary (1 month)	USA	39 (22.9)	Healthy, Black, 13 NC; 13 HC; 13 males	Y	Diary of symptoms	Diary number of drinks, absolute alcohol consumed (ounces)	No main effect of phase or group on consumption	
Holzhauer et al. (31)	Mixed (1 month)	USA	35 (19.9)	Healthy	Y	Countback method; diary of symptoms	Diary and CORE Alcohol and Drug Survey	No main effect of phase on consumption	Increase in consumption throughout FP if in a positive mood, More likely to drink in FP then LP if overall negative mood
Harvey and Beckman (32)	Diary (3 month)	USA	69 (24)	Healthy	–	Basal body temperature	Diary number of drinks, absolute alcohol consumed (ounces)	Consumption peaked once in the FP and once in the LP	
Holdstock and De Wit (33)	Lab (1 month)	USA	16 (25.3)	Healthy	–	Blood samples; ovulation test	Participants could choose up to three alcoholic drinks	No main effect of phase on consumption	
Mello et al. (34)	Lab (34 day)	USA	14 (26.6)	Healthy	–	Blood samples	Participants could choose between money or alcoholic beverages	No main effect of phase on consumption	5 increased alcohol during LP, 3 decreased alcohol use during LP; 6 did not differ; 4 increased from LP to FP; 4 decreased from LP to FP; 6 did not change
Pastor and Evans (35)	Diary (1 month)	USA	85 (25)	Healthy, 44 family history positive for AUD; 41 family history negative for AUD	–	Diary of symptoms	Diary number of drinks	More alcohol consumed during menses (FP) relative to premenses (LP) and postmenses (FP) for moderate drinkers. No effect of cycle phase on consumption for light or heavy drinkers.	
Pomerleau (36)	Diary (6 week)	USA	22 (30.5)	Healthy	–	Started participation in the same phase (FP)	Diary number of drinks	No main effect of phase on consumption	
Sutker et al. (37)	Diary (2 month)	USA	30 (NR)	Healthy, 7 NC; 12 HC; 11 male	Y	Blood samples, basal body temperature	Diary amount of alcohol consumed and % days drinking	No main effect of phase on consumption	NC drank to relieve tension during menses; HC drinking for pleasure more during corresponding menses; NC drank alone more during menses than other phases
Svikis et al. (38)	Diary (3 month)	USA	46 (32)	Healthy, 17 family history positive for AUD; 29 family history negative for AUD	–	Diary of symptoms	Diary of consumption	FH+ increase consumption from FP to LP, FH-decrease consumption from FP to LP	Correlation between craving and consumption for FH- group only
Tobin et al. (39)	Diary (3 month)	UK	37 (35)	Healthy, 21 PMS; 16 non-PMS	–	Diary of symptoms	Diary of consumption	No main effect of phase on consumption	Women with PMS had greater overall alcohol consumption
Warren et al. (40)	Lab (1 month)	UK	28 (20.2)	Healthy, 13 NC; 15 HC	Y	Menstrual cycle tracking application	mLs consumed	No main effect of phase or group on consumption	NC women craved alcohol more in the FP compared to the LP

*NR, not reported*.

* *included in analysis*.

### Diary Studies

#### Study Quality

Most studies described their method of determining menstrual cycle phase and the method of measuring alcohol consumption, with some using previously validated methods (25, 28–30, 32, 35, 36, 38, 39). However, generally the quality of the studies was poor, with issues relating to the lack of comparability between studies, and the absence of statistical results reported in full (25, 26, 28, 30, 36, 37, 39). Of those that did find a relationship, the specifics of this association differed as the studies reported different results with increased consumption in different phases. Interestingly, all six of the studies (25, 26, 29, 32, 35, 38) that found a statistically significant result had a sample size of over 40 participants (mdn = 69, range = 161), whereas only one (28) out of five of those that did not find a relationship had a sample size above 40 (mdn = 26, range = 45). It is possible that studies that did not detect a relationship were insufficiently powered, however most studies failed to report the effect sizes.

#### Study Descriptions and Findings

Some of the studies reported peaks in both phases, which could be related to other factors/characteristics. One (26) investigated phase-based alcohol consumption in relation to premenstrual syndrome (PMS) showed that participants consumed more alcohol in the LP (premenstrual) relative to the FP (post-menstrual; *p* < 0.05) whereas the control group (non-PMS) displayed the opposite pattern (*p* < 0.05).

Another study (32) defined five menstrual phases, finding a quadratic trend (*F* = 8.00, *df* = 1.66, *p* < 0.01) and a cubic trend (*F* = 6.65, *df* = 1.66, *p* < 0.05) with the quantity of alcohol consumption peaking in the mid-LP and again in the post-menstrual phase (FP).

Supporting peaks in both phases, another study (38) recorded the number of days bleeding [to determine the FP and premenstrual (LP)], symptoms, and number of drinks per day were recorded. They found a change in drinks per week from the FP to the LP (*F* = 10.05, *df* = 1.44, *p* = 0.003). Those Family History positive for AUD (FH+) showed an increase in consumption over this period, whereas the FH-group displayed a decreased in consumption. Additionally, in the FH-group, LP consumption was associated with increased alcohol craving (*F* = 13.73, *df* = 1.44, *p* = 0.0006). The final study finding peaks in both phases (25) aimed to investigate women's health across the menstrual cycle, including alcohol use found no overall effect of the menstrual cycle on alcohol use. However, gay women reported an increase in their alcohol consumption from the FP to LP (*p* < 0.05) whereas heterosexual women showed the opposite pattern (*p* < 0.05). They also reported significantly higher consumption in gay women and sexually abstinent women, compared with heterosexual women, during ovulatory and premenstrual (LP) phases (*p* < 0.05).

Two studies reported peaks in a single phase. One study (29) in Australia defined three groups of participants based on the Premenstrual Assessment Form (PAF; control, low-PMS, high-PMS). Self-reports monitored symptoms, bleeding days (used to determine cycle phases), and alcohol use. Those with PMS groups had higher alcohol consumption when premenstrual (LP) relative to post-menstrual (FP; *F* = 16.72, *df* = 1.40, *p* < 0.001). The other diary study (35) analyzed whether group (*n* = 85: light, moderate, and heavy drinkers) and/or menstrual cycle stage affected alcohol consumption using self-reported measures of alcohol consumption and cycle phase. The researchers reported that moderate drinkers showed an increase in alcohol consumed during menses (FP) relative to the premenstrual (LP) and post-menstrual (FP) phases (*ps* < 0.05).

Out of the five studies reporting no effect of the menstrual cycle on alcohol use, one (28) investigated the relationship across two groups of participants: high risk of AUD and low-risk of AUD based on family history of AUD. They found no significant effect of menstrual cycle phase on alcohol consumption.

One study (36) recruited 22 participants who all started participation at the same phase of their menstrual cycle [post-menstrual (FP)]. Participants recorded drinks per day and there were no significant differences across the cycle phases. In another study (39) the researchers examined alcohol consumption in women with PMS and controls. Researchers reported no significant effect of menstrual cycle phase on alcohol consumption, but women with PMS had greater overall alcohol use. Also recruiting separate group, one study (37) recruited three groups: naturally cycling, hormonal contraception, and males. Mean number of drinking days and amount of alcohol consumed for each phase did not differ. The final diary study which also included males (30) aimed to examine the effect of group and 5 cycle stages on consumption. It included three groups (*n* = 39: NC; HC; and males) where participants self-reported menstrual stage and alcohol consumption were recorded over one-month. The findings showed no effect of cycle stage on concumption. Nor did the researchers report a difference between the NC and HC groups.

Overall, eleven studies used diary methods for their data collection (25, 26, 28–30, 32, 35–39). Six of these studies found a relationship between menstrual cycle phase and alcohol consumption (25, 26, 29, 32, 35, 38). Of these, two reported the highest consumption to be in the premenstrual phase (LP) (26, 29) and one during menses [part of the FP; (35)]. One study reported a quadratic trend with two peaks in consumption once in the FP and the LP (32). Two studies showed mixed findings with consumption peaks depending on other factors: family history of alcohol use (38) and sexuality (25). The remaining five studies did not find a relationship between the menstrual cycle and alcohol use (28, 30, 36, 37, 39). The findings of the studies suggest that there may be a relationship between the menstrual cycle and alcohol use.

### Lab-Based Studies

#### Study Quality

The research in these studies were conducted within a controlled environment. However, although methods were included for determining sample size [e.g., power calculation (40)], overall the sample sizes were small and may have been unable to detect an association. As with the diary studies, generally the quality was poor, with the key quality issues relating to small sample sizes, unrealistic environments, and the absence of statistical results reported in full (33, 34, 40).

#### Studies' Descriptions and Findings

One study (34) reported that social drinkers showed different phase-based patterns of alcohol consumption; some increased consumption when premenstrual (LP) whereas others decreased consumption over the same period. This study was conducted within a research laboratory where participants could use credit to purchase alcoholic drinks.

Two studies reported no difference in consumption across the menstrual cycle. One study (33) gave participants three small doses of priming drink (0.2 g/kg) every half an hour and then offered up to three additional drinks. No significant differences were found across the phases for additional alcohol consumption.

The final lab study (40) used a menstrual cycle tracking application to determine cycle phase and measured alcohol consumption using a mock taste test with an alcoholic beverage. The researchers found no difference in alcohol consumption between menstrual cycle phases (*p* = 0.053) or across the groups (*p* = 0.440).

Overall, there were three studies that adopted lab-based methods. One study took place in a clinical residence (34) and the other two in a laboratory (33, 40). Both measured actual consumption without relying on self-report methods. However, the participants' consumption was capped in all studies to a set number of additional drinks. Additionally, two used blood sample analysis to determine cycle phase which is the most reliable method (33, 34) with the third using a menstrual tracking application (40). From these studies we cannot draw conclusions as the findings are mixed with no obvious reason for the inconsistencies.

### Mixed-Methods (Diary and Lab-Based) Studies

#### Study Quality

The final two studies adopted a mixed-methods approach (27, 31) and one included a sample of HC users (31). Both were conducted over one menstrual cycle and relied on self-report methods for measuring alcohol consumption, and used a previously validated method of determining cycle phase. As with all previous studies, there were key quality issues relating to small sample sizes and the absence of statistical results reported in full (e.g., exact *p*-values and/or effect sizes missing).

#### Studies' Descriptions and Findings

A recent study (31) investigated the effects of progesterone levels and menstrual cycle phase on alcohol use. The study included NC and HC groups. Participants attended the laboratory twice, completing a 2-week mood and alcohol diary between the visits. Saliva samples measured serum progesterone levels. There was no main effect of menstrual cycle phase on alcohol use, however, consumption was higher during the ovulatory phase compared to the menstrual phase for participants whose mood was more positive during the ovulatory phase.

Finally, a diary and lab-based study (27) investigated the effect of the menstrual cycle on alcohol use (*n* = 33) and impulsivity. Ovulation tests determined cycle phase, and alcohol use was self-reported. Although sensation seeking was influenced by menstrual cycle phase, alcohol consumption was not.

Together, neither study found any relationship between the menstrual cycle and alcohol consumption. The lab visits were included for different reasons: to collect samples (31) or measure CVC (27). Although both used previously validated methods, the small sample sizes make it difficult to draw conclusions, particularly as effect sizes were not reported.

### Overall Summary

Out of the 16 studies, 11 used diary methods (25, 26, 28–30, 32, 35–39) with self-reports used to measure alcohol consumption and menstrual cycle phase; three used laboratory-based methods (33, 34, 40), measuring actual alcohol consumption and using biological measures or applictions to determine cycle phase. The remaining two studies (27, 31) used mixed-methods with self-report data for alcohol consumption and biological measures for cycle phase. Three diary studies took place over one cycle (26, 30, 35) with the majority of the other studies collecting data for 2–3 months (28, 29, 32, 37–39). The lab and mixed-method studies all collected data over one cycle (27, 31, 33, 40, 41). The most important methodological consideration was sample size. Of the diary studies only seven out of the 11 had a sample size >40 (25, 26, 28, 29, 32, 35, 38), all lab studies had small samples [*n* < 29; (34, 37, 40)] as did the mixed-method studies (27, 31). Six out of the seven studies with larger samples reported a relationship between the menstrual cycle and alcohol consumption (25, 26, 29, 32, 35, 38). However, based on mixed findings the direction of the relationship remains inconclusive. As mentioned, most studies failed to report effect sizes which limits the interpretation of the results. Finally, some studies suggested any relationship would also depend on other factors, such as family history of AUD (38) and sexuality (25). An additional consideration is that the most recent studies were those that adopted mixed-methods (27, 31).

## Discussion

The primary aim of this systematic review was to explore whether there is an association between the phases of the menstrual cycle and alcohol use in human females, and whether hormonal contraception influences any associations. Overall, we cannot draw reliable findings from the studies in this systematic review to answer these questions. The current evidence, irrespective of the methods used, is inconsistent regarding whether the menstrual cycle influences alcohol use. Although a relationship was more common amongst studies with larger sample sizes, the sizes of these effects is largely unknown and must be considered in this interpretation. Of the studies that did find a relationship, conflicting findings suggested increased consumption in the FP, LP, or both. Additional findings suggest that there are multiple factors to consider (e.g., PMS, family history of AUD, sexuality). However, as there were a limited number of studies and the majority of these did not have the methodological rigor, our ability to draw firm conclusions is limited.

Consideration must be given to the fact that most analyses report two menstrual cycle phases and examination of any potential relationship requires consideration of all menstrual phases. Harvey and Beckman (32) showed participants peaked in consumption during the FP and the LP, which could be linked to the findings by Sutker and colleagues (37) who found that drinking motives differed with women drinking to relieve tension during the menstrual phase relative to the other phases. This is related to previous literature showing women are more likely to drink to cope than their male counterparts (42).

An additional aim of this review was to identify studies which have compared NC individuals to a HC comparison group. Only three studies did this (30, 37, 40), one reported that hormonal contraceptive users drank for pleasure which could be due to the steady levels of higher estrogens. Further supporting the potential role of estrogens in alcohol use, the researchers did find that motives differed within the naturally cycling group as they drank to relieve tension during the menstrual phase relative to the other phases. Warren and colleagues (40), however, found no effect of HC use on alcohol consumption in their lab-based study. Yet they also conducted a cross-sectional study which showed that HC users consumed more alcohol overall than NC individuals (40). Given that gonadal hormones may play a role in alcohol use, hormonal contraception (i.e., the combined oral contraceptive is made up of synthetic estrogens and synthetic progesterone) should be controlled for in studies exploring alcohol use, when testing female participants (6).

The studies included in this review highlighted that there are numerous other factors which should be taken into consideration when investigating the relationship between the menstrual cycle and alcohol use. For example, relationships between the menstrual cycle consumption and alcohol use are associated with a family history of AUD (38), PMS (26), sexuality (25), and mood state (31). Despite not finding a direct relationship between cycle phase and alcohol use, some studies found that factors known to influence drinking behavior (e.g., sensation seeking) were affected by menstrual cycle (27, 28, 33, 36, 37, 39). Premenstrual syndrome, which fluctuates over the cycle, is another factor for further consideration. Females with PMS tend to consume more alcohol overall compared to those who do not experience PMS (26, 39). Caan et al. (26) reported that those with PMS (mild-severe) were more likely to be heavy drinkers than the control group during the post-menstrual phase (FP). Future research would benefit from identifying the psychological mechanisms underlying alcohol use during different phases of the menstrual cycle. For example, mood is largely affected by both estrogens and PMS (43) and one of the studies found the relationship between phases and consumption to be dependent on mood (31), however, the underlying mechanism for this effect was not explored.

The final aim of this review was to provide methodological guidance for future research in this field. The literature presented display fundamental issues with regard to methodological rigor. Issues with sample size are problematic. Only one of the studies reported a sample size calculation (40), and it is possible the non-significant findings are partly due to insufficient power. This is further supported by the fact that other methodological differences seem not to have contributed to the conflicting results. Firstly, the length of time for participation varied between 1 and 3 months. For studies that were 1 month in duration there were five that reported an effect (25, 26, 31, 34, 35) and five that found no effect of the menstrual cycle on alcohol use (27, 30, 33, 36, 40). Secondly, regarding the determination of cycle phase, biological methods (blood/saliva samples, basal body temperature, and ovulation tests) were used in four studies that found an effect (25, 31, 32, 34) and three that did not find an effect (27, 33, 37). Of those using the countback method or logging days of menses/menstrual symptoms, three studies found an effect (29, 35, 38) and five did not (28, 30, 36, 39, 40). Finally, the methods for measuring alcohol use did not differ greatly between the studies. There were three lab studies measuring consumption, one which found an effect (34) and two that did not (33, 40). Similarly, two used recall of alcohol use, one found an effect (26) and the other did not (27). The remaining studies used self-report diary methods to measure alcohol consumption, six reported an effect (25, 29, 31, 32, 35, 38) and five did not (28, 30, 36, 37, 39). Future research should aim to ensure power calculations are used and sample sizes are adequate, and account for potential drop-out as most studies adopt a diary method which tend to have higher attrition.

The studies reported here also show different methods of menstrual cycle phase determination and alcohol use. More standardized methods of data collection are needed to allow for the comparison of findings. For example, menstrual cycle-based research often uses the countback method of determining cycle phase [self-reporting the dates of the previous menses to estimate phase (44)]. This method is an economic option, however, it may be beneficial to utilize menstrual cycle tracking applications to allow for more accurate predictions of phase (40). Additional methods (e.g., hormonal level analysis or basal body temperature) should also be used to confirm the phase (even if this is in a sub-sample to confirm other methods). Most of the studies presented included a within-subjects design, which future research should consider as the best method due to the variability of cycles both within and between females. In keeping with the current studies, future research should data collect for at least one whole cycle. Additionally, measures such as Ecological Momentary Assessment should be of utilized. These provide “real-world data” and can be preferred over retrospective self-report measures of alcohol consumption.

It is important for future research to comprehensively investigate the relationship between the menstrual cycle and alcohol use in order to inform healthcare professionals who work with females experiencing or recovering from alcohol misuse. This review encourages future research to disentangle how hormonal contraception affects alcohol use in relation to the menstrual cycle in order to inform such treatments.

To conclude, the present systematic review suggests that the association between menstrual cycle phases and alcohol use is inconclusive, as is the role of hormonal contraception in this association. This is due to existing literature reporting different findings, with no particular methodologies appearing superior. There is now a need for the comprehensive investigation of the effect of menstrual cycle and hormonal contraception on alcohol use with larger sample sizes, which includes potential influencing factors known to affect both.

## Data Availability Statement

The original contributions presented in the study are included in the article/supplementary material, further inquiries can be directed to the corresponding author/s.

## Author Contributions

JW, AR, LG, and SG contributed to conception and design of the review. JW and VF conducted the review searches and data extraction. JW, VF, and AR synthesized the data. JW wrote the first draft of the manuscript. All authors contributed to manuscript revision, read, and approved the submitted version.

## Funding

The studies reported were in part funded by the John Lennon Memorial Scholarship, this funding body had no influence over the research conducted or the publication process.

## Conflict of Interest

The authors declare that the research was conducted in the absence of any commercial or financial relationships that could be construed as a potential conflict of interest.

## Publisher's Note

All claims expressed in this article are solely those of the authors and do not necessarily represent those of their affiliated organizations, or those of the publisher, the editors and the reviewers. Any product that may be evaluated in this article, or claim that may be made by its manufacturer, is not guaranteed or endorsed by the publisher.

## Appendix

### Scoping Search

A scoping search was conducted during October–December 2018. From the research question and inclusion/exclusion criteria, the initial search strategy was (“Menstrua^*^” OR “Menses” OR “Period” OR “Menorrhea”) AND “Alcohol^*^” AND NOT “pregnan^*^” AND NOT (“irregul^*^ OR abnormal^*^”) AND NOT (“disorder” OR “problem” OR “abuse”) AND NOT “steroid^*^.” The primary aim was to yield studies with a NC group and HC control group, as such “Contraception” was not an exclusive search term. From the scoping search there were 1,210 titles after duplicates were removed. JGW reviewed abstracts from titles deemed relevant to refine the search terms.

After reviewing the search results the following terms were removed as they yielded either irrelevant topics i.e., “Period” yielded results relating to time in subject areas such as history. This term was replaced with “Menarche” as a synonym for the menstrual cycle. Additionally, from the results it was determined that additional terms for alcohol were needed i.e., “ethanol” and “drink^*^.” As some studies compared those with abnormal menstrual cycles to healthy females we removed the exclusion terms “irregul^*^” and “abnormal^*^.” Similarly the terms “disorder,” “problem,” and “abuse” were removed as again some studies had healthy control groups. Finally, steroid^*^ was removed as an exclusion as the term did not appear often enough in the titles.
